# Neurological Adverse Events Associated With Esketamine: A Disproportionality Analysis for Signal Detection Leveraging the FDA Adverse Event Reporting System

**DOI:** 10.3389/fphar.2022.849758

**Published:** 2022-04-08

**Authors:** Haoning Guo, Bin Wang, Shuying Yuan, Silin Wu, Jing Liu, Miaoquan He, Jisheng Wang

**Affiliations:** ^1^ Division of Psychopharmacology, Department of Pharmacy, The Third Hospital of Mianyang Sichuan Mental Health Center, Mianyang, China; ^2^ Clinical Experimental Center, Jiangmen Central Hospital, Affiliated Jiangmen Hospital of Sun Yat-sen University, Jiangmen, China; ^3^ Department of Pathology, The Third Hospital of Mianyang, Sichuan Mental Health Center, Mianyang, China

**Keywords:** esketamine, pharmacovigilance, neurological adverse events, signal, FAERS, disproportionality analysis

## Abstract

Esketamine was approved for the treatment of treatment-resistant depression in 2019. After the approval of esketamine, numerous concerns have been raised regarding its long-term safety and tolerability. A previous systematic pharmacovigilance study on esketamine-related adverse events (AEs) was published in 2020; however, it has not been updated 2 years later. The primary aim of this study was to detect and characterize neurological safety signals of esketamine to partially update the knowledge in this field using the FDA pharmacovigilance database. Reporting odds ratio (ROR) was calculated for esketamine-related neurological AEs from 2019 to 2021 with a signal considered when the lower limit of the 95% confidence interval (CI) of ROR (ROR_025_) exceeded one. Severe and non-severe cases were compared using an independent samples *t*-test or chi-squared (χ2) test, and a rating scale was used to prioritize the signals. The database contained 720 cases of esketamine-associated neurological AEs, with 21 signals detected, ranging from a ROR_025_ of 1.05 (disturbance in attention) to 204.00 (sedation). 16 latest neurological AEs emerged in the second year of marketing approval of esketamine, with eight signals detected. The associations between esketamine and nervous system disorders persisted when stratifying by sex, age, and reporter type, whereas the spectrum of neurological AEs differed in stratification regimens. Esketamine dosage, antidepressant polypharmacy, or co-prescription with benzodiazepines affected AEs severity (*t* = 2.41, *p =* 0.017; χ2 = 6.75, *p =* 0.009; and χ2 = 4.10, *p* = 0.043; respectively), while age and sex did not (*p* = 0.053 and *p* = 0.397, respectively). Three signals were categorized as moderate clinical priority [i.e., sedation, dizziness, and dysgeusia (priority points 7, 5, and 5, respectively)], showing the same early failure type profiles. Notably, seven detected disproportionality signals were not previously detected in clinical trials. Although the majority of results were in line with those obtained in the previous study, there were discrepancies in the spectrum of neurological AEs and the effects of several risk factors on AEs severity among the two studies that should be recognized and managed early in clinical treatments.

## Introduction

The United States (U.S.) Food and Drug Administration (FDA) and European Medicines Agency approved esketamine plus an oral antidepressant for treatment of treatment-resistant depression (TRD) in adults in 2019 (Kim et al., 2019; Schatzberg, 2019; U.S. Food and Drug Administration, 2019; Horowitz and Moncrieff, 2020; Psychopharmacologic Drugs Advisory Committee, 2020) based primarily on two pivotal positive phase 3 trials (Daly et al., 2019; Popova et al., 2019) that demonstrated a statistically significantly larger reduction in the Montgomery–Asberg Depression Rating Scale scores and decrease in the risk of relapse. Soon after, the authorities expanded the indication of esketamine to include patients with major depressive disorder with acute suicidal behaviors or ideation (McIntyre et al., 2021). Esketamine, a non-selective, non-competitive N-methyl-D-aspartate (NMDA) receptor antagonist, is the *S*-enantiomer of racemic ketamine (Bozymski et al., 2020; Janssen Inc, 2020). By antagonizing the NMDA receptor, esketamine transiently increases glutamate release, resulting in increased stimulation of α-amino-3-hydroxy-5-methyl-4-isoxazolepropionic acid receptors, with this stimulation augmenting neurotrophic signaling and possibly facilitating the restoration of synaptic function in brain areas responsible for the adjustment of mood and emotional behavior (Janssen Inc, 2020). However, the exact mechanisms by which esketamine exerts its antidepressant effects remains unclear.

After the approval of esketamine, numerous concerns have been raised regarding its long-term safety and tolerability. A previous systematic pharmacovigilance study of esketamine-associated AEs was published in August 2020 and included all reports recorded in the FDA Adverse Event Reporting System (FAERS) from the second quarter (Q2) of 2019–2020 Q1 (Gastaldon et al., 2020). This real-world study detected a range of new, unexpected signals and identified several risk factors related to AEs severity, such as the female, patients treated with higher doses, and those receiving multidrug therapy (Gastaldon et al., 2020). Because esketamine has been on the market for >2 years, it would be instructive for clinicians and pharmacovigilance experts to be presented what has changed 1 year after the publication of a similar article. Additionally, it remains unclear whether the risk factors identified in the previous study would be reliable predictors of the outcomes of AEs in a specific system organ class. Two recent meta-analyses reveal that, in general, neurological AEs are among the most commonly reported ones in patients following treatment with esketamine (Zheng et al., 2020; Wang et al., 2021). Also, many esketamine-associated neurological side effects emerged in the previous pharmacovigilance study (Gastaldon et al., 2020). On these grounds, the characteristics of neurological AEs associated with esketamine are the special focus in this study.

In the present study, we analyzed the safety data of esketamine from the FAERS database in order to detect and characterize relevant neurological safety signals. We then compared the main results of these two pharmacovigilance studies in order to identify new findings in the present study and partially update the current knowledge in this field. Additionally, we performed stratification analyses, clinical prioritization of signals, and time-to-onset analysis in order to further investigate the properties of esketamine-related neurological AEs.

## Materials and Methods

### Study Design and Data Sources

A disproportionality analysis reflecting the case/non-case study design, was used to quantify the associations between esketamine and neurological AEs. This measures the occurrence of target AEs associated with a drug compared to all other drugs in the database. If the proportion of a target AE is larger in the suspected group of drugs (case) than in the non-exposed group (non-case), an association is hypothesized to exist between the drug and the AE and conceived as a disproportionality signal (Gastaldon et al., 2020). Because esketamine was approved for marketing by the U.S. FDA in March 2019, this study included all reports from the first quarter (Q1) of 2019–2021 Q2.

### Data Extraction and Descriptive Analysis

**FIGURE 1:**
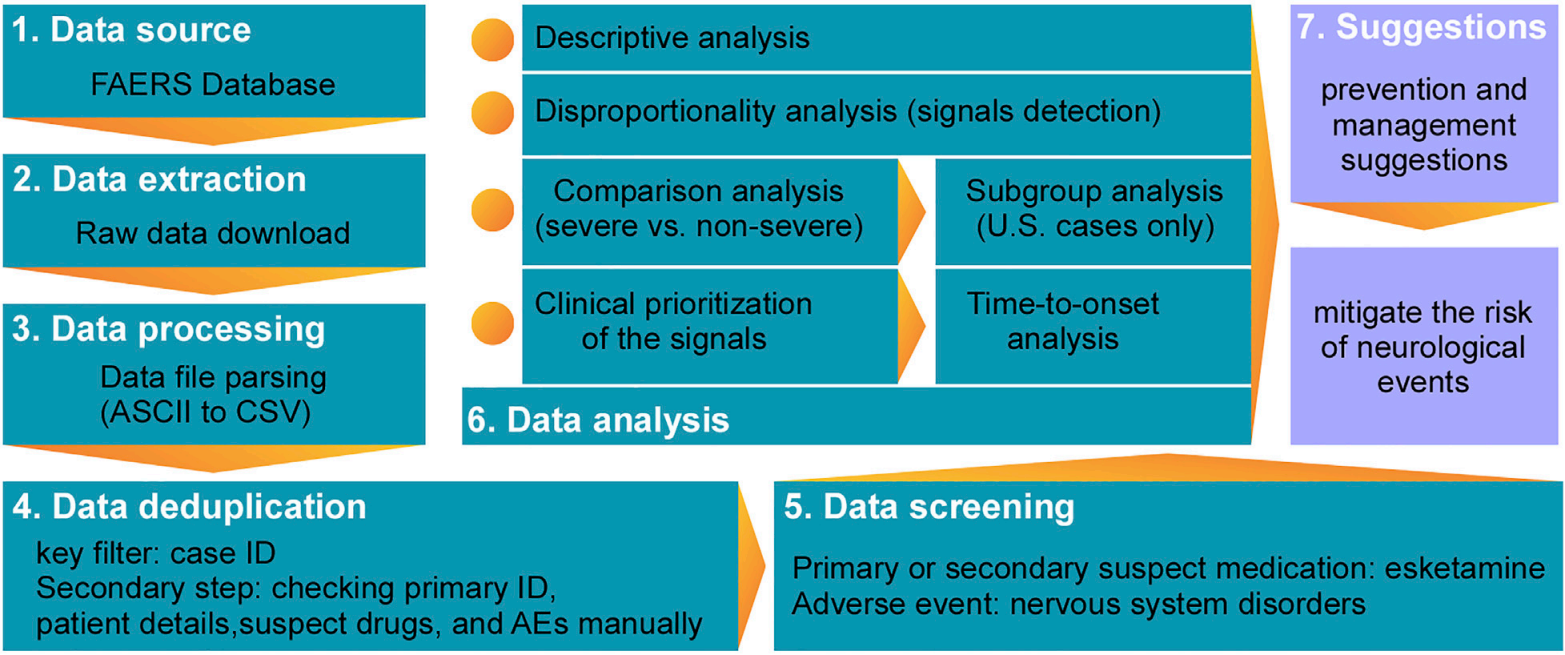
Multi-step process of data extraction, processing, and analysis.

Seven data files, including patient demographics and administrative information, drug/biologic information, the Medical Dictionary for Regulatory Activities (MedDRA) terms for AEs, patient outcomes, report sources, drug therapy start/end dates, and MedDRA terms for diagnoses/indications, were downloaded from the FAERS database (https://fis.fda.gov/extensions/FPD-QDE-FAERS/FPD-QDE-FAERS.html#collapse2020) and processed using Golang (v1.15; http://go.dev/doc/go1.15). Because FAERS may sporadically include duplicate reports submitted by various sources, duplicates were identified and removed accordingly, as described in previous studies (Carnovale et al., 2018; Gastaldon et al., 2020; Mazhar et al., 2021). The case ID was chosen as the key filter in this study to remove duplicate records. We further reviewed the records manually according to the similarities of primary ID, patient details (e.g., age and sex), suspect drugs, and AEs when the case ID was the same. Only reports with the latest FDA received date were selected, and duplicate records were removed accordingly. All reports recorded in FAERS with esketamine considered as the primary or secondary suspect medication were eligible for inclusion in the analysis. This study included all nervous system disorders according to the MedDRA terminology (v23.0), with all of these events coded on the preferred term (PT) level to identify or select specific symptoms or signs of neurological entities. We ultimately retrieved and described detailed information, including patient characteristics (sex, age at onset, and weight), general information (reporter region, reporting year, and reporter qualification), drugs (dosage regimen), reactions (reported terms, MedDRA classification terms, and event date), seriousness (serious and non-serious), final outcome, and concomitant drugs. Means (± standard deviation) were used to characterize continuous normally distributed variables and proportions for categorical variables. A flowchart reporting the multi-step process of data extraction, processing, and analysis is shown in Figure 1.

### Statistical Analysis

We calculated the reporting odds ratio (ROR) for all selected AEs with at least five reports in order to reduce the likelihood of false positives (Raschi et al., 2017). ROR is a disproportionality approach widely applied based on the principles of calculations using a 2 × 2 table (Supplementary Table S1) (Salem et al., 2018). The 95% confidence interval (CI) was estimated for the ROR, and a signal was considered when the lower limit of the 95% CI of the ROR (ROR_025_) exceeded one.

Previous findings report that several factors, such as patient physical and mental status, concomitant medicines, pharmaceutical formulation, route of drug administration, and disease severity, may impact the occurrence and severity of AEs associated with esketamine or ketamine (Ceban et al., 2021; McIntyre et al., 2021). However, no biomarkers or phenomenological features have been proven to be reliable predictors of outcome with ketamine in patients with TRD (McIntyre et al., 2021). We then compared the severe and non-severe reports to clarify the severity of the detected safety signals and identify risk factors in patients. AEs were classified as serious or non-serious, with serious cases defined as death, a life-threatening adverse drug experience, inpatient hospitalization or prolongation of existing hospitalization, a persistent or severe disability, a congenital anomaly/birth defect, as well as other serious medical events (Johnson et al., 2019). We compared age, weight, sex, esketamine dose, AE types, and concomitant drugs between serious and non-serious reports. Because reports from countries outside the U.S. are also been collected in the FAERS database, we further performed a subgroup analysis by including U.S. cases only in order to reduce the impact of geographic variation in AE reporting. Proportions were compared using a Pearson’s chi-squared (χ2) or Fisher’s exact test, and an independent samples *t* test was applied for continuous data, such as age and dose. Data were analyzed using SPSS (v22.0; IBM Corp., Armonk, NY, United States), and statistical significance was set at a two-tailed *p* < 0.05.

Finally, we conducted stratification analysis by sex (female and male), age (18–64 and ≥65 years), and reporter type (healthcare professionals and consumers) separately to further explore the impact of different stratification regimens on the associations between esketamine and nervous system disorders.

### Clinical Prioritization of Signals

AEs with disproportionality signals are generally classified into three types according to the level of clinical importance: 1) weak clinical priority, 2) moderate clinical priority, and 3) strong clinical priority (Gatti et al., 2020). We applied a scale to prioritize signals in five aspects; 1) number of target AEs, 2) ROR_025_ values, 3) mortality proportion, 4) assessment as important medical events (IMEs) or designated medical events (DMEs), and 5) results of current evidence evaluation (Gatti et al., 2020). The detailed information is shown in Supplementary Table S2.

### Time-to-Onset Analysis

Time-to-onset (TTO) of a given event was calculated as the difference between the start of treatment and the date the event occurred. Analyses of TTO data were performed based on median duration, quartiles, and the Weibull shape parameter (WSP) test (Mazhar et al., 2021), which is used to determine the varying rate of incidence of AEs (Mazhar et al., 2021). The scale parameter α and shape parameter β are used to describe the Weibull distribution (Kinoshita et al., 2020; Mazhar et al., 2021). We calculated the median TTO and WSP of signals with moderate or strong clinical priority after the beginning of the esketamine therapy in order to predict the hazard of the occurrence of these AEs over time. The selection of parameters and criteria for evaluation were described in previous studies (Kinoshita et al., 2020; Mazhar et al., 2021). All WSP tests were conducted using Minitab statistical software (v19.1.0; Minitab LLC, State College, PA, United States).

## Results

### Descriptive Analysis

**FIGURE 2:**
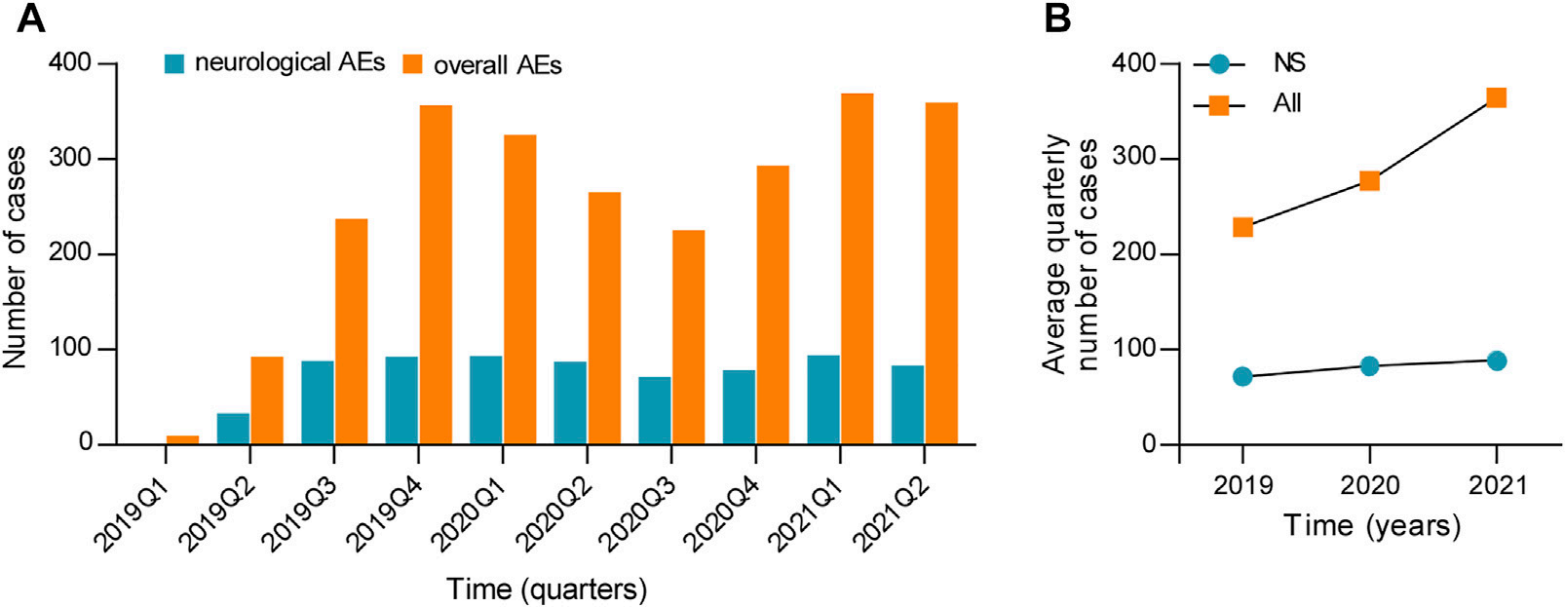
**(A)** Number of cases and **(B)** the average quarterly number of cases for neurological and overall AEs associated with esketamine. NS, nervous system; AEs, adverse events.

A total of 5,592,554 individual cases of AEs have been submitted to the FAERS database since 2019 and containing 993 esketamine-related neurological AEs in 720 patients. The detailed clinical characteristics of patients with esketamine-induced AEs are listed in Table 1. Serious cases of neurological and overall AEs, including six (1.28%) and 87 (6.06%) deaths, were recorded in 420 and 1,282 patients, respectively. Other serious events and hospitalization were the most frequently reported severe outcomes and occurred in 443 (94.25%) and 1,265 (88.15%) cases of neurological and overall events, respectively. There were 214 (29.72%), 329 (45.69%), and 177 (24.58%) neurological reports received in 2019, 2020, and the first half of 2021, respectively. The detailed quarterly numbers of submitted cases for neurological and overall AEs are shown in Figure 2A. Age data were available for 519 patients (mean age: 46.59 ± 15.32 years); notably, no patient was <18 years of age. Neurological AEs predominantly affected females (64.01%), with a female to male sex ratio of 1.78:1. Nervous system AEs reports were submitted by healthcare professionals and consumers in 616 (85.67%) and 103 (14.33%) cases, respectively.

**TABLE 1:** Clinical characteristics of patients with esketamine-induced adverse events.

	Esketamine induced neurological AEs		Esketamine induced overall AEs	
	Available number	Value	Available number	Value
Sex, n (%)	639 (88.75)	—	2044 (80.79)	—
Female, n (%)	—	409 (64.01)	—	1,279 (62.57)
Male, n (%)	—	230 (35.99)	—	765 (37.43)
Age at onset, n (%)	519 (72.08)	—	1,386 (54.78)	—
Mean ± SD, years	—	46.59 ± 15.32	—	47.56 ± 14.99
<18, n (%)	—	0 (0.00)	—	3 (0.22)
18–64, n (%)	—	455 (87.67)	—	1,199 (86.50)
≥65, n (%)	—	64 (12.33)	—	184 (13.28)
Reporting year, n (%)	720 (100)	—	2,530 (100)	—
2021 Q1-2	—	177 (24.58)	—	728 (28.77)
2020	—	329 (45.69)	—	1,108 (43.80)
2019	—	214 (29.72)	—	694 (27.43)
Reporters, n (%)	719 (99.86)		2,529 (99.96)	
Healthcare professional	—	616 (85.67)	—	2081 (82.29)
Consumer	—	103 (14.33)	—	448 (17.71)
Reporter region, n (%)	720 (100)	—	2,530 (100)	—
U.S.	—	644 (89.44)	—	2,198 (86.88)
Outside U.S.	—	76 (10.56)	—	332 (13.12)
Patient weight, n (%)	153 (21.25)	—	520 (20.55)	—
Mean ± SD, kg	—	81.35 ± 26.78	—	82.20 ± 24.13
Outcomes, n (%)	720 (100)	—	2,530 (100)	—
Non-serious		300 (41.67)	—	1,248 (49.32)
Serious cases	—	420 (58.33)	—	1,282 (50.68)
Died	—	6 (1.28)	—	87 (6.06)
Dissabled	—	3 (0.64)	—	13 (0.91)
Hospitalized	—	90 (19.15)	—	474 (33.03)
Life threatening	—	18 (3.83)	—	70 (4.88)
Other outcomes	—	353 (75.10)	—	791 (55.12)

AEs, Adverse Events; n, number of cases.

### Disproportionality Signals

**FIGURE 3:**
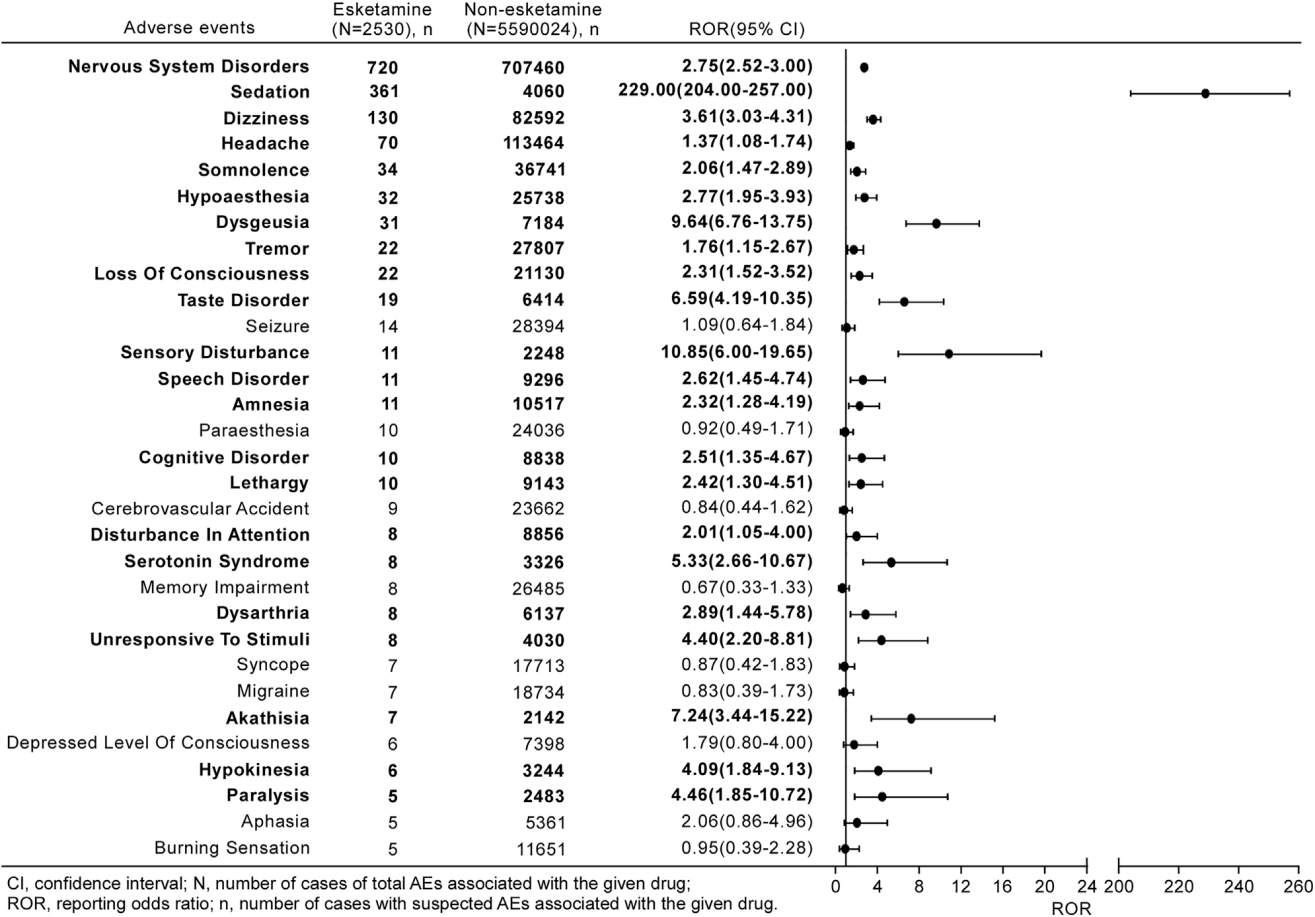
Reporting odds ratios (ROR) with 95% CI for all esketamine-related neurological AEs with at least five counts. Results that are statistically significant are in bold.

From 2019 to 2021, 90 different PTs related to neurological AEs after receiving esketamine were reported in the FAERS database, 30 of which were mentioned in at least five reports (Figure 3). The three most commonly reported neurological events were sedation (*n* = 361, 36.35%), dizziness (*n* = 130, 13.09%), and headache (*n* = 70, 7.05%).

Figure 3 shows a full list of disproportionality results for the esketamine-associated neurological AEs occurring in at least five reports. The frequency of the reported nervous system disorders with esketamine was strikingly higher than that for non-esketamine in the entire database, with an ROR_025_ of 2.52. Further investigation of the subgroups revealed 21 neurological signals after esketamine treatment, with values of signals ranging from a ROR_025_ of 1.05 (disturbance in attention) to 204.00 (sedation). Other events, including paresthesia, memory impairment, and cerebrovascular accidents, were not over-reported in this population.

### Serious Versus Non-Serious Cases

Esketamine dose differed statistically significantly between severe and non-severe cases of neurological AEs (69.88 ± 15.46 mg vs. 63.93 ± 15.54 mg, respectively; *p* = 0.017) (Table 2). By contrast, weight and age did not differ between the two groups (*p* = 0.521 and *p* = 0.053, respectively). Additionally, a higher proportion of males exhibited serious AEs than females; however, the difference was not statistically significant (χ2 = 0.72, *p* = 0.397). Sedation (χ2 = 31.15, *p* < 0.001), loss of consciousness (χ2 = 14.49, *p* < 0.001), and serotonin syndrome (*p* = 0.024) were more likely to be reported as serious AEs, whereas dizziness (χ2 = 4.12, *p* = 0.042), dysgeusia (χ2 = 7.98, *p* = 0.005), taste disorders (χ2 = 9.64, *p* = 0.002), and cognitive disorders (*p* = 0.002) were more likely to be reported as non-serious AEs. Patients with severe AEs were more likely to receive combination therapy with antidepressant polypharmacy (χ2 = 6.75, *p* = 0.009), benzodiazepines (χ2 = 4.10, *p* = 0.043), or somatic medications (χ2 = 5.05, *p* = 0.025) than patients with non-serious AEs. After assessing U.S. cases only, risk factors related to the severity of neurological AEs persisted. Detailed results are shown in Supplementary Table S3.

**TABLE 2:** Differences in clinical characteristics of severe and non-severe reports.

	Serious cases	Non-serious cases	Statistic	*p* value
Age, years (Mean ± SD)	47.37 ± 14.90	44.40 ± 16.34	1.94^e^	0.053
weight, kg (Mean ± SD)	80.19 ± 23.28	83.04 ± 31.53	0.64^e^	0.521
Sex distribution	—	—	—	—
Female, n (%)	257 (62.84)	152 (37.16)	0.72^f^	0.397^c^
Male, n (%)	153 (66.52)	77 (33.48)		
Esketamine dose, mg	69.88 ± 15.46	63.93 ± 15.54	2.41^e^	0.017
Mean ± SD				
Types of AEs, n (%)	—	—	—	—
Sedation	248 (59.05)	113 (37.67)	31.15^f^	＜0.001^c^
Dizziness	65 (15.48)	65 (21.67)	4.12^f^	0.042^c^
Headache	38 (9.05)	32 (10.67)	0.36^f^	0.552^c^
Somnolence	20 (4.76)	14 (4.67)	0.01^f^	0.905^c^
Hypoaesthesia	18 (4.29)	14 (4.67)	0.01^f^	0.951^c^
Dysgeusia	10 (2.38)	21 (7.00)	7.98^f^	0.005^c^
Tremor	9 (2.14)	13 (4.33)	2.14^f^	0.143^c^
Loss of consciousness	22 (5.24)	0 (0.00)	14.49^f^	＜0.001^c^
Taste disorder	4 (0.95)	15 (5.00)	9.64^f^	0.002^c^
Sensory disturbance	10 (2.38)	1 (0.33)	—	0.057^d^
Speech disorder	8 (1.90)	3 (1.00)	—	0.376^d^
Amnesia	4 (0.95)	7 (2.33)	—	0.216^d^
Cognitive disorder	1 (0.24)	9 (3.00)	—	0.002^d^
Lethargy	7 (1.67)	3 (1.00)	—	0.534^d^
Disturbance in attention	4 (0.95)	4 (1.33)	—	0.725^d^
Serotonin syndrome	8 (1.90)	0 (0.00)	—	0.024^d^
Dysarthria	6 (1.43)	2 (0.67)	—	0.480^d^
Unresponsive to stimuli	7 (1.67)	1 (0.33)	—	0.149^d^
Akathisia	2 (0.48)	5 (1.67)	—	0.135^d^
Hypokinesia	3 (0.71)	3 (1.00)	—	0.697^d^
Paralysis	5 (1.19)	0 (0.00)	—	0.079^d^
Antidepressant polypharmacy^a^	65 (15.48)	26 (8.67)	6.75^f^	0.009^c^
Concomitant drugs	553	208	—	—
Mood stabilizers	18 (3.25)	8 (3.85)	0.03^f^	0.860^c^
Hypnotics	94 (17.00)	34 (16.35)	0.01^f^	0.916^c^
Benzodiazepines	74 (78.72)	20 (58.82)	4.10^f^	0.043^c^
Antipsychotics	54 (9.76)	23 (11.06)	0.15^f^	0.695^c^
Somatic medications^b^	281 (50.81)	86 (41.35)	5.05^f^	0.025^c^
Opioids	10 (3.56)	2 (2.33)	—	0.740^d^
Others	106 (19.17)	57 (27.40)	—	—

The AEs, listed above were AEs, with disproportionality signal.

a Antidepressant polypharmacy in the table defined as at least two antidepressants apart from esketamine in a report.

b Somatic medications in the table defined as co-prescription antihypertensive, analgesic, lipid-lowering agents etc.

c Proportions were compared using Pearson χ2 test.

d Fisher’s exact test.

e The t-statistic of the independent samples *t* test.

f The χ2 statistic of the Pearson chi-square test.

AEs, Adverse Events; n, number of cases.

### Stratification Analysis

**FIGURE 4:**
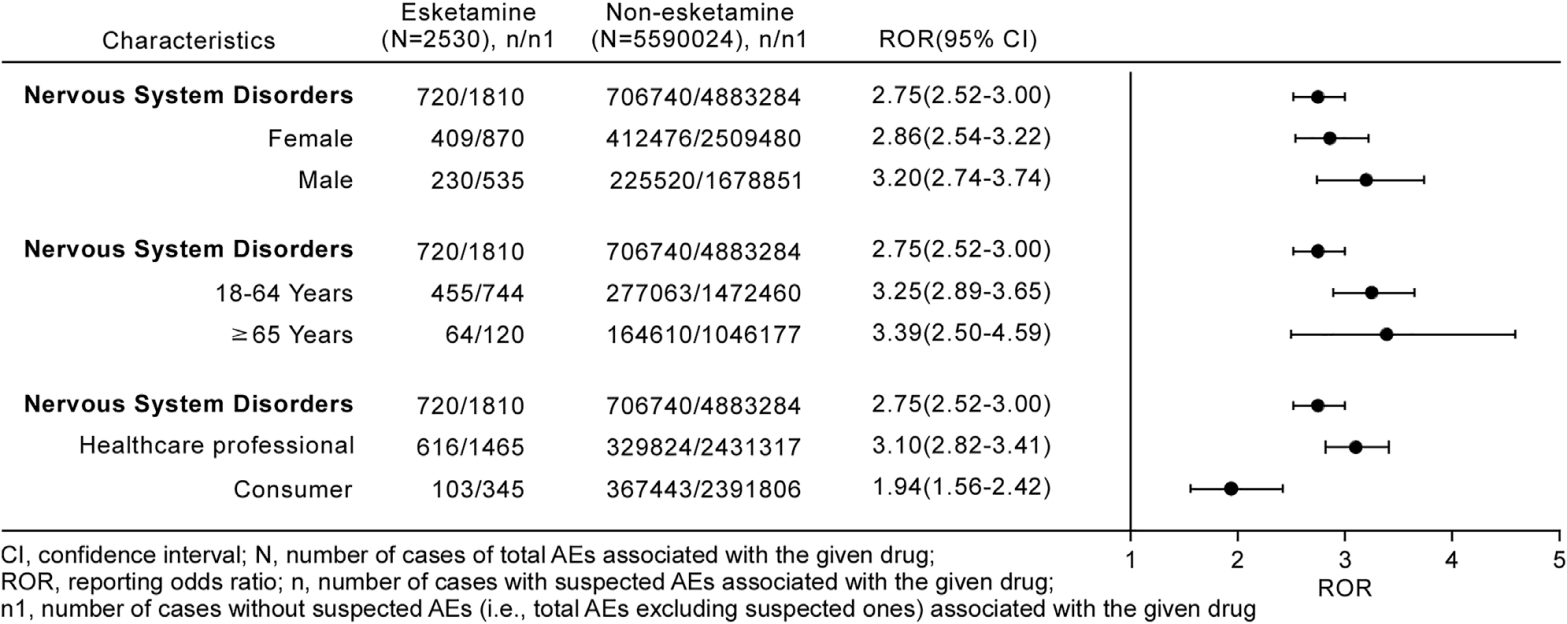
Stratification analysis of esketamine-induced nervous system disorders.

**FIGURE 5:**
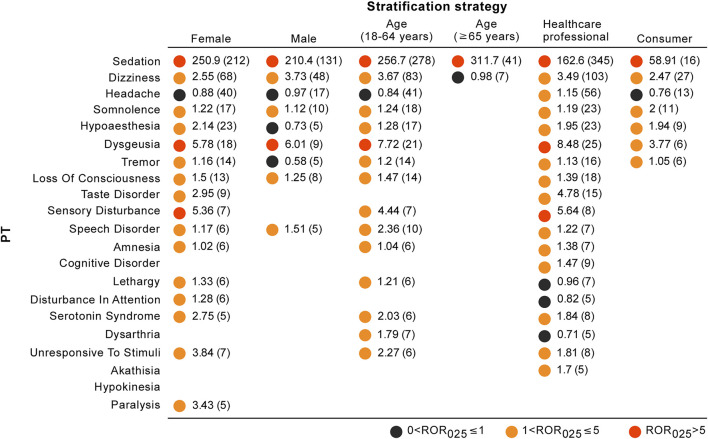
Neurological toxicity spectrums for different stratification strategies. The results are expressed in the form of ROR_025_ (n). Blank spaces represent not eligible for disproportionality analysis (AEs with at least 5 reports were eligible for analysis).

We used three different stratification strategies to increase the robustness of the findings. After separately assessing nervous system disorders stratifying by sex, age, and reporter type (Figure 4), the ROR_025_ values of all stratification subgroups were greater than one and the associations between esketamine and nervous system disorders persisted. However, the spectrum of neurological AEs differed in stratification regimens, with the detailed stratification analysis results shown in Figure 5.

### Clinical Prioritization of the Signals

In total, 10 of the 21 PTs (47.62%) with disproportionality signals were categorized as IMEs, whereas none represented DMEs (Table 3). According to the clinical priority assessment results, 18 (85.71%), three (14.29%), and zero PTs were graded as weak, moderate, and strong clinical priority, respectively. Sedation (*n* = 361; ROR_025_ = 204.00; and priority score = 7), dizziness (*n* = 130; ROR_025_ = 3.03; and priority score = 5), and dysgeusia (*n* = 31; ROR_025_ = 6.76; and priority score = 5) were considered moderate clinical priorities. In the assessment of the relevant evidence, 10 PTs showed a strong level of evidence, and seven detected disproportionality signals were not previously detected in clinical trials (i.e., sensory disturbance, amnesia, cognitive disorder, serotonin syndrome, akathisia, hypokinesia, and paralysis).

**TABLE 3:** Clinical priority assessing results of disproportionality signals.

PTs	n	ROR_025_	Death (n)	IMEs/DMEs	Relevant evidence evaluation	Priority level (score)
Sedation	361	204.00	4	IME	++	Moderate (7)
Dizziness	130	3.03	2	NA	++	Moderate (5)
Headache	70	1.08	0	NA	++	Weak (4)
Somnolence	34	1.47	0	IME	++	Weak (4)
Hypoaesthesia	32	1.95	0	NA	++	Weak (3)
Dysgeusia	31	6.76	0	NA	++	Moderate (5)
Loss of consciousness	22	1.52	0	IME	+	Weak (3)
Tremor	22	1.15	0	NA	++	Weak (3)
Taste disorder	19	4.19	0	NA	+	Weak (3)
Speech disorder	11	1.45	0	IME	+	Weak (3)
Sensory disturbance	11	6.00	1	IME	−	Weak (4)
Amnesia	11	1.28	0	NA	−	Weak (1)
Lethargy	10	1.30	0	IME	++	Weak (4)
Cognitive disorder	10	1.35	0	NA	−	Weak (1)
Serotonin syndrome	8	2.66	0	NA	−	Weak (1)
Dysarthria	8	1.44	0	IME	++	Weak (3)
Disturbance in attention	8	1.05	0	IME	++	Weak (3)
Unresponsive to stimuli	8	2.20	0	IME	+	Weak (3)
Akathisia	7	3.44	0	NA	−	Weak (1)
Hypokinesia	6	1.84	0	NA	−	Weak (0)
Paralysis	5	1.85	0	IME	−	Weak (1)

A priority score between 8 and 10, 5–7 or 0–4 represents the signal with strong, moderate or weak clinical priority, respectively.

NA, Not Applicable (for relevant criterias); n, number of cases; PTs, Preferred Terms; ROR_025_, the lower limit of 95% confidence interval of ROR.

### Time-to-Onset Analysis

Results of TTO and WSP analyses for sedation, dizziness, and dysgeusia are summarized in Table 4. The median TTO of sedation, dizziness, and dysgeusia associated with esketamine was 1.00 (range: 0–621), 0.00 (range: 0–433), and 0.00 (range: 0–17) days, respectively. In the WSP test for sedation, both the shape parameter β and the upper limit of its 95% CI were <1, suggesting an early failure type, with the same failure type identified for dizziness and dysgeusia.

**TABLE 4:** The results of time-to-onset analysis for signals with moderate prioritization.

Adverse events	—			Weibull distribution				Failure type
	Cases	TTO (days)		Scale parameter		Shape parameter		
	n	Median (IQR)	Min-max	α	95% CI	β	95% CI	
Sedation	177	1.00 (0.00–27.00)	0–621	5.11	2.80–9.32	0.28	0.24–0.34	Early failure
Dizziness	66	0.00 (0.00–29.50)	0–433	4.21	1.36–13.09	0.25	0.19–0.34	Early failure
Dysgeusia	8	0.00 (0.00–0.00)	0–17	0.01	0.00–777.83	0.16	0.03–0.89	Early failure

n, number of cases with available time-to-onset; IQR, interquartile range; TTO, Time-to-onset. When TTO, is 0 days, the adverse event occurred within the same day with the therapy.

## Discussion

This report provides the most updated findings linking esketamine with the neurological safety profiles based on the real-world population. Although the majority of results were in line with those obtained in the previous study, there were discrepancies in the spectrum of neurological AEs and the effects of several risk factors on AEs severity among the two studies. Overall, several major findings emerged that deserve further discussion.

### The Increasing Trend for Submitting Esketamine Reports to Food and Drug Administration Adverse Event Reporting System

Because esketamine was approved in March 2019, the number of neurological and overall AEs was low in 2019 Q1, with only one (0.14%) and nine (0.36%) cases, respectively. Therefore, we calculated the average number of quarterly reports by excluding data from 2019 Q1 in order to more precisely compare the annual growth trend of esketamine safety reports (Figure 2B). Figure 2B shows that there has been a marked increase in the mean number of esketamine reports per quarter from 2019 to 2020, with the annual total counts in 2020 almost 1.6-fold higher than that in 2019. This growth trend of esketamine reports across this time window is similar to the results reported in the preceding study, which indicated that the increasing trend was noteworthy, and that the number of monthly esketamine-associated AEs had nearly doubled in 2020 relative to 2019 (Gastaldon et al., 2020). Additionally, the authors predicted that the number of esketamine reports might expand further in the next year (Gastaldon et al., 2020). The present findings confirm this prediction, with a higher increase in the average number of quarterly reports in 2021 compared to 2020 (Figure 2B). Assuming that the Weber effect is correct [i.e., the reporting peak happened 2 years after approval of the drug (Hoffman et al., 2014)], we propose the hypothesis that esketamine reports may gradually stabilize from 2021 Q3 onwards.

We then evaluated the increasing trend of esketamine-related neurological AEs. The counts of neurological safety reports increased in the first 9 months of 2019 and have exhibited a relatively stable trend thereafter (Figure 2A). In summary, although we argue that notoriety bias [e.g., substantially increased AEs reporting stimulated by media attention (Khouri et al., 2021)] has no marked impact on the reporting pattern of esketamine-related neurological AEs, an enhanced post-marketing safety surveillance remains necessary.

### Comparison of Safety Signals Between Two Pharmacovigilance Studies

Generally, disproportionality analysis cannot be used as an independent method to assess drug-related risks in real-world populations or replace formal clinical trials owing to its inability to quantify the incidence rates of adverse events. However, recent findings suggest that there is a significant correlation with the strength of association between disproportionality analyses and risk estimates in clinical studies (e.g., relative risks) (Van Puijenbroek et al., 2002; Maciá-Martínez et al., 2016; Khouri et al., 2021). Therefore, in the absence of data from clinical trials or epidemiological studies, disproportionality analysis provides at least an important indication of the prioritization of AEs and helps in the design of future studies (Maciá-Martínez et al., 2016; Khouri et al., 2021). Although the results for a majority of signals were in line with those obtained in the previous study, there were discrepancies in the spectrum of esketamine-associated neurological AEs between the two studies. One year after esketamine approval, 18 esketamine-related neurological AEs with at least four reports were registered in the FAERS database (Gastaldon et al., 2020), whereas in the present study, the total number increased to 34, representing 16 newly recorded AEs emerged in the following year. The full list of all new esketamine-relateded neurological AEs is provided in Supplementary Table S4. Further analysis of these new AEs detected eight signals, including amnesia (ROR_025_ = 1.28), loss of consciousness (ROR_025_ = 1.52), paralysis (ROR_025_ = 1.85), serotonin syndrome (ROR_025_ = 2.66), disturbance in attention (ROR_025_ = 1.05), hypertonia (ROR_025_ = 4.63), nystagmus (ROR_025_ = 3.97), and unresponsive to stimuli (ROR_025_ = 2.20). Interestingly, three events, i.e., cognitive disorder, headache, and tremor were less frequently reported in the previous study (Gastaldon et al., 2020) than in our study (ROR_025_ were respectively 0.89 vs. 1.35, 0.75 vs. 1.08, and 0.86 vs. 1.15 in the previous study vs. our analysis). In summary, the spectrum of safety signals may change over time as more reports are submitted in the future. Healthcare professionals should continuously monitor medication safety and ensure timely reporting of any AEs to spontaneous reporting systems.

The present study showed the three most commonly reported neurological events were sedation, dizziness, and headache. In particular, sedation was the only signal related to all stratification regimens (Figure 5) and more likely to be reported as a severe AE. Similarly, sedation was the signal with the highest ROR_025_ values among all neurological AEs in both previous pharmacovigilance study (Gastaldon et al., 2020) and the present analysis, stimulating our interest to further explore its essential features. We extracted a total of 361 (50.14%) sedation cases, with 248 (68.70%) eventually reported as serious cases, including six deaths. These results are consistent with those reported in a series of randomized controlled studies, where sedation was substantially more frequent in the esketamine groups (49–61%) than in the placebo-treated groups (10–19%), with a relative risk of up to 4.75 (Fedgchin et al., 2019; Popova et al., 2019; Ochs-Ross et al., 2020; Zheng et al., 2020). Moreover, other clinical characteristics of sedation warrant further attention. In the TTO analysis, the median TTO of sedation was 1 day, implying that at least half of the patients developed a rapid (within 24 h) onset of sedation. This also accords with previous clinical trials, which demonstrated that the latest onset and resolution time of any degree of sedation among all participants were 90 and 210 min after esketamine use, respectively (Fedgchin et al., 2019; Popova et al., 2019; Ochs-Ross et al., 2020; Psychopharmacologic Drugs Advisory Committee, 2020). Furthermore, several subjects experienced marked fluctuations in the way sedation occurred, indicating that the post-dose times of onset, peak, resolution, and severity varied among follow-up visits and suggesting that previous experience cannot accurately predict the future onset pattern (Fedgchin et al., 2019; Popova et al., 2019; Ochs-Ross et al., 2020; Psychopharmacologic Drugs Advisory Committee, 2020). Therefore, due to the potential severity and fluctuating mode of sedation, clinicians should monitor patients for at least 2-h post-dose to alleviate excessive sedation risk (e.g., motor vehicle accidents, falls) associated with esketamine.

### Serious Versus Non-Serious Reports

Of particular interest to clinicians and patients is whether they would be more or less likely to be safe to benefit from ketamine or esketamine management from an *a prior*i perspective (McIntyre et al., 2021). The present analysis indicated that esketamine dosage, antidepressant polypharmacy, or co-prescription with benzodiazepines but not age or sex may correlate with an increased risk of AEs severity. The descriptive analysis in Table 1 showed that females (64.01%) were more inclined to report neurological AEs, and further comparison of severe and non-severe cases revealed that although the proportion of serious AEs was numerically higher in males than in females (66.52 vs. 62.84%), the two groups did not differ statistically. This result is slightly different from the finding of a previous pharmacovigilance study that suggested women were at a higher risk of reporting severe AEs after receiving esketamine (Gastaldon et al., 2020). Few studies have evaluated the effect of sex on esketamine-related side effects; however, some previous observations provided evidence of the differential effects of sex on AE occurrence in patients receiving the esketamine parent compound ketamine (Winstock et al., 2012; Zhang et al., 2013; Chen et al., 2014). An observational study found that male patients receiving ketamine were more likely to undergo psychotic disorders (Zhang et al., 2013), and other studies revealed that cognitive impairment mainly affected female ketamine users (Zhang et al., 2013; Chen et al., 2014). By contrast, Winstock et al. (2012) noted an absence of any association with sex in urinary symptoms among ketamine patients. Additionally, the present stratification analysis showed that the spectrum of neurological AEs differed between females and males, with females associated with more neurological event categories than males (Figure 5). The probable mechanisms of distinct sex effects on ketamine-related toxicities remain unclear, although several explanations have been proposed, such as sex hormones and pharmacokinetic properties (Lee et al., 2000; Zarate et al., 2012; Gastaldon et al., 2020). Although no general conclusions have been reached on the exact effects of sex on esketamine-induced toxicity, sex should be viewed as a key factor in clinical treatment and future research. In the previous study, Gastaldon et al. (2020) compared age between reports with serious and non-serious outcomes and found that there was no statistical difference in age among two groups (*p* = 0.807), which is consistent with the finding in our current study. Additionally, we performed stratification analysis by age (18–64 and ≥65 years) and found that the ROR_025_ values of nervous system disorders were similar among these two subgroups (2.89 and 2.50, respectively) and two disproportionality signals were present (Figure 4). Therefore, the associations between esketamine and nervous system disorders persisted when stratifying by age. However, this study observed the spectrum of neurological AEs differed markedly between stratification regimens by age, and patients aged ≥65 years reported fewer neurological event categories than those <65 years (Figure 5). This is consistent with recent studies indicating that the common AEs were reported less frequently in patients ≥65 years of age than in those <65 years after esketamine use (Bozymski et al., 2020; Ochs-Ross et al., 2020; Psychopharmacologic Drugs Advisory Committee, 2020). However, our results should be interpreted with caution and require further investigations for verification based on the low reporting cases among geriatric patients.

The present study revealed that patients taking a higher dose of esketamine were more prone to develop serious neurological toxicity. A possible explanation for this might be that the increases in Cmax and area under the receiver operating characteristic curve were nearly dose-proportional when the dose of esketamine was increased from 56 to 84 mg, with a subsequent increase in the risk of dose-dependent adverse reactions (U.S. Food and Drug Administration, 2019; McIntyre et al., 2021). Other possible high-risk factors were the intake of combination therapy with antidepressant polypharmacy, somatic medications, or benzodiazepines, potentially due to channeling bias (e.g., selectively co-prescribing other drugs with esketamine to patients with more severe conditions) and potential drug–drug interactions affecting treatment outcomes (Gastaldon et al., 2020; Raschi et al., 2018; Carmona-Huerta et al., 2019). The above findings support the conclusions of another study in this field, with the exception of patients receiving co-medication with antipsychotics or mood stabilizers (Gastaldon et al., 2020). Moreover, we noticed that benzodiazepines were more tend to be administered concomitantly with esketamine than other hypnotics in the reported cases (Table 2). However, the concomitant use of benzodiazepines increased the risk of developing severe neurological AEs (χ2 = 4.10, *p* = 0.043). This finding is consistent with those of previous studies, which suggested that concomitant esketamine use with central nervous system depressants (e.g., benzodiazepines, opioids, alcohol) may worsen sedation (U.S. Food and Drug Administration, 2019; Diekamp et al., 2021). Given the frequent need for co-prescriptions of hypnotics with esketamine, we recommend that non-benzodiazepines (e.g., zolpidem, zopiclone, zaleplon and eszopiclone) should be administered to mitigate the risk of severe neurological events.

### Clinical Prioritization of the Signals

In recent years, there has been an exponential increase in research on disproportionality analysis, particularly concerning the rapid detection of safety signals for recently approved drugs (Raschi et al., 2018). In the present study, we sought to advance the application of our disproportionality analysis by utilizing a rating scale in order to prioritize safety signals and avoid unnecessary warnings. This approach may also be beneficial in helping clinicians and pharmacovigilance researchers improve the reliability of disproportionality signals by evaluating the current evidence. The disproportionality signals with moderate clinical priority other than sedation were dizziness and dysgeusia. Dizziness is among the most commonly observed adverse reactions in patients being treated with esketamine and usually manifests as exertion dizziness, postural dizziness, and procedural dizziness. A pooled analysis of completed phase 3 studies found that dizziness was less tolerable in the esketamine group than in the placebo group (27.6–33.0% vs. 6.4–9.7%) (Fedgchin et al., 2019; Popova et al., 2019; Ochs-Ross et al., 2020), with an odds ratio (OR) of 4.47 (95% CI: 3.27–6.11, *p* < 0.0001) (Wang et al., 2021). Additionally, in the TTO analysis, the median TTO of dizziness was 0 days, with an early failure type profile, suggesting that most patients would report dizziness within the same day of esketamine treatment, and then the risk of dizziness occurrence gradually decreased over time. However, the data on time-to-onset for dizziness slightly contrasted what is known from the previous pharmacovigilance study, showing dizziness with a mean time-to-onset of 15.7 days (Gastaldon et al., 2020). This discrepancy mainly resulted from the different computation methods of TTO data. According to a recent meta-analysis of controlled trials, dysgeusia was statistically significantly more commonly reported in the intranasal esketamine arm than in the placebo arm (OR = 1.67, 95% CI: 1.21–2.31; *p* = 0.022) (Wang et al., 2021). Furthermore, the frequency of dysgeusia in various age ranges differs, and patients aged <65 years appear to be associated with relatively higher dysgeusia frequency than patients aged  ≥65 years (18.8 vs. 5.6%) (Fedgchin et al., 2019; Popova et al., 2019; Ochs-Ross et al., 2020), which further supports the findings of our stratification analysis (number of dysgeusia reports: 21 vs. 2, respectively) (Figure 5).

Seven unexpected AEs with a detected signal require further attention in the future, including sensory disturbance, amnesia, cognitive disorder, serotonin syndrome, akathisia, hypokinesia, and paralysis. These events were not previously detected in pre-marketing pivotal clinical trials and only reported in the pharmacovigilance database. Surprisingly, an unexpected finding was the absence of convincing clinical evidence for the association between esketamine and cognitive disorder. In a phase I study that recruited healthy volunteers (Morrison et al., 2018), a single dose of 84 mg esketamine caused statistically significant cognitive performance impairment at 40-min post-dose versus placebo-treated participants (all five tests, *p* < 0.005). By contrast, cognitive performance on these tests was comparable between the esketamine- and placebo-treated groups at 2-, 4-, or 6-h post-dose (Morrison et al., 2018). Additionally, the CogState Computerized Test Battery used to assess cognition performance in phase 3 studies revealed no statistically significant differences between the placebo and esketamine groups, except for findings in the SUSTAIN-2 trial (this trial provided evidence of slowing of the reaction time in elderly participants) (Daly et al., 2019; Fedgchin et al., 2019; Popova et al., 2019; Ochs-Ross et al., 2020; Wajs et al., 2020). Nevertheless, it remains difficult to distinguish the drug effect from other factors because of the high degree of intra-individual variability in the SUSTAIN-2 trial (Wajs et al., 2020). Although no universally convincing conclusions have been drawn on the exact impact of esketamine on cognitive function, cognitive impairment is still listed in the warning and precaution section of the instruction manual (U.S. Food and Drug Administration, 2019), likely owing to the fact that cognitive and memory impairments have been reported with long-term ketamine use or abuse (Na and Kim, 2021). Consequently, the exact effects of esketamine on cognition and the mechanisms of this potential association require further investigation.

### Prevention and Management Suggestions

Clinical trials and several systematic reviews report that neurological disorders occur in ∼20–60% of patients following administration (Caddy et al., 2015; Kishimoto et al., 2016; Fedgchin et al., 2019; Janssen Inc, 2020; Xiong et al., 2021). Therefore, there is a strong need for effective preventive and management measures for these side effects in order to ensure greater acceptability and effectiveness of the treatments. Preventive interventions (e.g., establishing a comfortable therapeutic environment without over-stimulation, playing soothing music, and instruction to perform breathing and mindfulness exercises) and constant patient surveillance are the primary methods for alleviating neurological side effects (Ceban et al., 2021). Additionally, patient evaluation (e.g., prescribing esketamine to patients already at high risk should be done with extreme caution), patient education and instruction, and skillful care operations are crucial steps in mitigating adverse reactions (Ceban et al., 2021; McIntyre et al., 2021). Furthermore, clinicians need to fully consider the necessity of adjunctive medications [e.g., moderate-to-severe headaches can be addressed using analgesics, such as acetaminophen (Ceban et al., 2021); and benzodiazepines may worsen sedation and are not recommended], dosage reduction [e.g., in cases of dose-dependent neurological AEs, such as sedation, the dose of esketamine should be decreased to promote tolerability, although the efficacy may be compromised (Psychopharmacologic Drugs Advisory Committee, 2020; McIntyre et al., 2021)], and suspension or discontinuation of therapy owing to serious adverse reactions.

### Limitations and Strengths

Notably, the findings of this study based on the FAERS database need to be interpreted with caution, in consideration of several limitations shared by all pharmacovigilance databases, including the possibility of submitting incomplete, inaccurate, untimely, and unverified information (Hauben et al., 2007; Salem et al., 2018; Caldito et al., 2021). Additionally, the incidence rates of AEs cannot be calculated by disproportionality analysis due to the lack of the total size of the population using esketamine and could also be affected by over- or under-reporting (Gastaldon et al., 2020). Furthermore, the establishment of causality between a drug and AE is also restricted in pharmacovigilance studies (Salem et al., 2018). Another limitation of the present study is that we focused on the AEs in one reaction group alone, so the generalization between our findings and other system organ classes is unknown. Notwithstanding such limitations, the new findings in the present study could potentially prompt improved awareness of esketamine-related toxicities and provide some support evidence to confirm the conclusions found by Gastaldon et al. (2020). Finally, spontaneous individual safety reports provide a valuable source of drug safety information and remain the cornerstone for post-marketing safety assessments.

## Conclusion

To the best of our knowledge, this report provides the most updated analysis linking esketamine with nervous system safety profile based on a larger number of individual safety reports. One of the main findings is that 16 latest neurological AEs emerged in the second year of marketing approval of esketamine, with eight signals detected. Moreover, we observed a continuously increasing trend of esketamine overall reports in the period from 2019 to 2021 and proposed a hypothesis that esketamine reports may gradually stabilize from the second half of 2021 onwards. However, the growth trend in neurological AEs is somewhat different, exhibiting a relatively stable trend since 2019 Q4. A higher dose of esketamine, antidepressant polypharmacy, and combination treatment with benzodiazepines or somatic medications are more likely to be risk factors related to AEs severity, whereas age and sex are not. Three AEs with moderate signal prioritization exhibit the same early failure type profiles, suggesting that the risk of these AEs occurrence associated with esketamine increases at an earlier stage of the treatment. From a prospective viewpoint, given the growing use of esketamine, pharmacovigilance studies will likely play a central role in facilitating risk–benefit assessment through vigorous long-term monitoring, particularly for unanticipated AEs. In summary, our findings and management recommendations could potentially prompt improved awareness of esketamine-related toxicities and help clinicians/researchers mitigate the risk of neurological events. In the meantime, more intensive studies on other systemic organ classes are warranted in the future to comprehensively examine the safety profiles of esketamine.

## Data Availability

The original contributions presented in the study are included in the article/Supplementary Material, further inquiries can be directed to the corresponding authors.
