# Corrigendum: Neurological adverse events associated with esketamine: A disproportionality analysis for signal detection leveraging the FDA adverse event reporting system

**DOI:** 10.3389/fphar.2022.1075966

**Published:** 2022-11-09

**Authors:** Haoning Guo, Bin Wang, Shuying Yuan, Silin Wu, Jing Liu, Miaoquan He, Jisheng Wang

**Affiliations:** ^1^ Division of Psychopharmacology, Department of Pharmacy, The Third Hospital of Mianyang, Sichuan Mental Health Center, Mianyang, China; ^2^ Clinical Experimental Center, Jiangmen Central Hospital, Affiliated Jiangmen Hospital of Sun Yat-Sen University, Jiangmen, China; ^3^ Department of Pathology, The Third Hospital of Mianyang, Sichuan Mental Health Center, Mianyang, China

**Keywords:** esketamine, pharmacovigilance, neurological adverse events, signal, FAERS, disproportionality analysis

In the original article, there was an error in affiliation **1**. Instead of “Division of Psychopharmacology, Department of Pharmacy, The Third People’s Hospital of Mianyang, Sichuan Mental Health Center, Mianyang, China”, it should be “Division of Psychopharmacology, Department of Pharmacy, The Third Hospital of Mianyang, Sichuan Mental Health Center, Mianyang, China”. In the published article, there was an error in affiliation **3**. Instead of “Department of Pathology, The Third People’s Hospital of Mianyang, Sichuan Mental Health Center, Mianyang, China”, it should be “Department of Pathology, The Third Hospital of Mianyang, Sichuan Mental Health Center, Mianyang, China”.

The authors apologize for this error and state that this does not change the scientific conclusions of the article in any way. The original article has been updated.

