# Supplementary material for: Neurological Adverse Events Associated With Esketamine: A Disproportionality Analysis for Signal Detection Leveraging the FDA Adverse Event Reporting System
Source: Front Pharmacol. 2022 Apr 8;13:849758. doi: 10.3389/fphar.2022.849758 (PMC9023790; doi:10.3389/fphar.2022.849758)
Supplement: Supplementary file 1 [file DataSheet1.docx]

***Supplementary Material***

**Neurological Adverse Events Associated with Esketamine: A Disproportionality Analysis for Signal Detection Leveraging the FDA Adverse Event Reporting System**

**SUPPLEMENTARY TABLE 1** Calculation of reporting odds ratio (ROR).

|  | Cases with suspected AE  (e.g., sedation) | Cases without suspected AE  (e.g., all AEs excluding sedation) |
| --- | --- | --- |
| Cases with esketamine | A | B |
| Cases without esketamine  (e.g., entire database) | C | D |

A: Number of cases with suspected AE (e.g., sedation) associated with esketamine. B: Number of cases without suspected AE (e.g., all AEs excluding sedation) associated with esketamine. C: Number of cases with suspected AE (e.g., sedation) associated with all other drugs in entire database. D: Number of cases without suspected AE (e.g., all AEs excluding sedation) associated with all other drugs in entire database.

AEs, Adverse Events; ROR, Reporting Odds Ratio; CI, confidence interval.

The calculation formulas are shown below:

$ROR=\frac{A/B}{C/D}=\frac{\mathrm{AD}}{\mathrm{BC}}$ (1)

$95 \% CI=e^{\ln\left( \mathrm{ROR} \right) \pm1.96\sqrt{\frac{1}{A}+\frac{1}{B}+\frac{1}{C}+\frac{1}{D}}}$ (2)

**SUPPLEMENTARY TABLE 2** A rating scale assessing clinical priority of disproportionality signals.

| **Assessment items** | **2 points** | **1 point** | **0 point** |
| --- | --- | --- | --- |
| Number of target events | ＞50 | 10-50 | ＜10 |
| ROR_025_ | ＞5 | 2-5 | 1-2 |
| Mortality proportion | ＞50% | 25-50% | ＜25% |
| IMEs or DMEs | DME | IME | None |
| Current evidence evaluation | ++ | + | **-** |

Mortality proportion: percentage of cases in which death was reported as an outcome in the overall cases report for a particular adverse event. IMEs and DMEs are developed and updated by EMA (European Medicines Agency, 2020). ++ : AEs are mainly from the FDA Prescribing Information, the Summary of Product Characteristics of esketamine posted by the MHRA, Phase 2/3 RCTs, or systematic reviews, with biological plausibility. + : AEs are mainly from other clinical trials, observational studies, or case reports/series with potential biological plausibility. - : AEs only emerging from disproportionality analyses.

AEs, Adverse Events; DMEs, Designated Medical Events; IMEs, Important Medical Events; MHRA, Medicine and Healthcare Products Regulatory Agency; RCTs, Randomized Controlled Trials; ROR_025_, the lower limit of 95% confidence interval of ROR.

**SUPPLEMENTARY TABLE 3** A subgroup analysis of severe and non-severe reports.

| **Characteristics** | **Serious cases** | **Non-Serious** | **Statistic** | ***p* value** |
| --- | --- | --- | --- | --- |
| **Age, years (**Mean ± SD) | 47.10±14.86 | 44.47±16.52 | 1.66^e^ | 0.098 |
| **Weight, Kg (**Mean ± SD) | 85.96±25.11 | 83.00±31.79 | 0.58^e^ | 0.566 |
| **Esketamine dose, mg**  **(**Mean ± SD) | 70.14±15.72 | 63.92±14.91 | 2.36^e^ | 0.020 |
| **Sex distribution**, n (%) |  |  |  |  |
| female | 209 | 149 | 1.12 ^f^ | 0.290 ^c^ |
| male | 131 | 76 |  |  |
| **Antidepressant polypharmacy**^a^ | 54(15.52) | 26(8.78) | 6.06^f^ | 0.014 ^c^ |
| **Concomitant drugs** | 505 | 208 |  |  |
| mood stabilizers | 15(2.97) | 8(3.85) | 0.14^f^ | 0.712 ^c^ |
| hypnotics | 93(18.42) | 34(16.35) | 0.30^f^ | 0.583 ^c^ |
| benzodiazepines | 73(78.49) | 20(58.82) | 3.96^f^ | 0.047 ^c^ |
| antipsychotics | 48(9.50) | 23(11.06) | 0.24^f^ | 0.623 ^c^ |
| somatic medications^b^ | 261(51.68) | 86(41.35) | 5.89^f^ | 0.015 ^c^ |
| opioids | 9(3.45) | 2(2.33) |  | 0.999 ^d^ |
| others | 88(17.43) | 57(27.40) |  |  |
| ^a^Antidepressant polypharmacy in the table defined as at least two antidepressants apart from esketamine in a report. ^b^Somatic medications in the table defined as co-prescription antihypertensive, analgesic, lipid-lowering agents and etc. ^c^Proportions were compared using Pearson χ2 test. ^d^Fisher’s exact test. ^e^The t value of the independent samples t test. ^f^The χ2 value of the Pearson chi-square test.  n, number of cases. | | | | |

**SUPPLEMENTARY TABLE 4** Newly recorded neurological AEs with at least four reports in the second year of marketing approval of esketamine.

| **Adverse events** | **n** | **ROR** | **Lower 95% CI** | **Upper 96% CI** |
| --- | --- | --- | --- | --- |
| **amnesia** | **11** | **2.32** | **1.28** | **4.19** |
| **loss of consciousness** | **22** | **2.31** | **1.52** | **3.52** |
| seizure | 14 | 1.09 | 0.64 | 1.84 |
| **disturbance in attention** | **8** | **2.01** | **1.05** | **4.00** |
| memory impairment | 8 | 0.67 | 0.33 | 1.33 |
| **serotonin syndrome** | **8** | **5.33** | **2.66** | **10.67** |
| **unresponsive to stimuli** | **8** | **4.40** | **2.20** | **8.81** |
| migraine | 7 | 0.83 | 0.39 | 1.73 |
| syncope | 7 | 0.87 | 0.42 | 1.83 |
| depressed level of consciousness | 6 | 1.79 | 0.80 | 4.00 |
| aphasia | 5 | 2.06 | 0.86 | 4.96 |
| burning sensation | 5 | 0.95 | 0.39 | 2.28 |
| **paralysis** | **5** | **4.46** | **1.85** | **10.72** |
| **hypertonia** | **4** | **12.40** | **4.63** | **33.25** |
| **nystagmus** | **4** | **10.63** | **3.97** | **28.49** |
| psychomotor hyperactivity | 4 | 1.48 | 0.37 | 5.94 |
| Adverse events, AEs; n, number of cases; ROR, reporting odds ratio; CI, confidence interval. Results that are statistically significant are in bold. | | | | |

**References**

European Medicines Agency. (2020). Inclusion/exclusion criteria for the “Important Medical Events” list. 2019. Available at: <https://wwwemaeuropaeu/en/documents/other/eudravigilance-inclusion/exclusion-criteria-important-medical-events-list_enpdf> (Accessed Nov 1, 2019)
